# Pathological findings after third- and second-generation everolimus-eluting stent implantations in coronary arteries from autopsy cases and an atherosclerotic porcine model

**DOI:** 10.1038/s41598-021-85740-2

**Published:** 2021-03-18

**Authors:** Suguru Migita, Daisuke Kitano, Yuxin Li, Yutaka Koyama, Sayaka Shimodai-Yamada, Akira Onishi, Daiichiro Fuchimoto, Shunichi Suzuki, Yoshiyuki Nakamura, Taka-aki Matsuyama, Seiichi Hirota, Masashi Sakuma, Masahiko Tsujimoto, Atsushi Hirayama, Yasuo Okumura, Hiroyuki Hao

**Affiliations:** 1 Division of Human Pathology, Department of Pathology and Microbiology, Nihon University School of Medicine, 30-1 Oyaguchi-kamicho, Itabashi-ku, Tokyo 173-8610 Japan; 2 Division of Cardiology, Department of Medicine, Nihon University School of Medicine, Tokyo, Japan; 3 Division of Cell Regeneration and Transplantation, Department of Functional Morphology, Nihon University School of Medicine, 30-1 Oyaguchi-kamicho, Itabashi-ku, Tokyo 173-8610 Japan; 4 Department of Animal Science and Resources, College of Bioresource Sciences, Nihon University, Kanagawa, Japan; 5 Institute of Agrobiological Sciences, National Agriculture and Food Research Organization (NARO), Ibaraki, Japan; 6 Swine and Poultry Research, Saitama Prefectural Agricultural Technology Research Center, Saitama, Japan; 7 Department of Legal Medicine, Showa University School of Medicine, Tokyo, Japan; 8 Department of Surgical Pathology, Hyogo College of Medicine, Nishinomiya, Japan; 9 Department of Cardiovascular Medicine, Dokkyo Medical University School of Medicine, Tochigi, Japan; 10 Department of Pathology, Osaka Police Hospital, Osaka, Japan; 11 Department of Cardiology, Osaka Police Hospital, Osaka, Japan

**Keywords:** Drug discovery, Cardiology, Medical research

## Abstract

Pathological changes after third-generation drug-eluting stent implantation remain unclear. We compared the tissue responses of coronary arteries after the implantation of third-generation abluminal biodegradable-polymer everolimus-eluting stent (3rd EES) and second-generation durable-polymer EES (2nd EES) using autopsy specimens and an atherosclerotic porcine model. We compared the histology of stented coronary arteries obtained by autopsy performed 1–10 months after 3rd EES (n (number of cases) = 4, stent-implanted period of 3–7 months) and 2nd EES (n (number of cases) = 9, stent-implanted period of 1–10 months) implantations. The ratio of covered stent struts was higher with 3rd EESs than with 2nd EESs (3rd; 0.824 ± 0.032 vs. 2nd; 0.736 ± 0.022, *p* = 0.035). Low-density lipoprotein receptor knockout minipigs were stented with 3rd or 2nd EES in the coronary arteries and the stented regions were investigated. The fibrin deposition around the 2nd EES was more prominent. Additionally, higher density of smooth muscle cells was confirmed after the 3rd EES implantation. Pathological examination after the 3rd EES demonstrated a combination of less fibrin deposition and more rapid acquisition of well-developed neointima as compared to the 2nd EES at autopsy and the atherosclerotic porcine model.

## Introduction

Percutaneous coronary intervention techniques have advanced markedly in recent decades. First-generation durable-polymer drug-eluting stents (1st DESs), have reduced the incidence of target lesion revascularization which was occasionally seen in patients treated with bare metal stents^1,2^. However, ratios of stent thrombosis (ST) in the late post-implantation period (> 1 year), which have been referred to as very late stent thrombosis (VLST), were higher with 1st DESs than with bare metal stents^1,2^. Pathological examination of patients who died because of ST after 1st DES implantation revealed delayed arterial healing^3,4^.

In addition to the adoption of dual antiplatelet therapy for prevention of ST, second-generation durable-polymer drug-eluting stents (2nd DESs) have been developed. They consist of a thin strut platform coated with further biocompatible durable copolymers and limus-based drugs.

Clinical trials have shown similar efficacy and better long-term safety of 2nd DES, as compared with 1st DES^5–12^. Additionally, there are reports claiming amelioration of the pathology associated with 2nd DES^13,14^.

However, atherosclerotic neointima, referred to as “neoatherosclerosis”, and subsequent VLST due to neointimal rupture have both been observed^15^. To reduce the risk of such complications, third-generation biodegradable-polymer drug-eluting stents (3rd DESs) have been developed. These stents were designed to have thinner struts, improved deliverability, and biodegradable polymers. Additionally, except for some 3rd DESs, most of them have abluminal polymers allowing only the strut surface, which is in contact with the vessel wall, to be coated with drugs.

Several clinical studies have demonstrated the superiority of 3rd DESs to 2nd DESs^8,16–19^.

Because of the improved design and positive clinical reports^20,21^, 2nd DESs are still widely used throughout the world. Although several clinical studies have demonstrated the improved effectiveness of 3rd DESs, the underlying pathological response is poorly understood and merits further investigation.

In the present study, we examined the initial tissue responses of human coronary arteries within 1 year after 3rd-generation abluminal biodegradable-polymer everolimus-eluting stents (3rd EESs) or 2nd-generation durable-polymer everolimus-eluting stents (2nd EESs) implantation using autopsy specimens. In addition, in order to evaluate the tissue responses of coronary arteries over time after EES implantations, we also studied an experimental animal model allowing assessment of matched conditions.

Coronary arteries of wild type pigs have been used to assess the efficacy and safety of stents. However, the vascular responses of healthy coronary arteries from wild type pigs are markedly different from those of atherosclerotic coronary arteries in patients with ischemic heart diseases. Our group previously bred low-density lipoprotein receptor knockout (LDLR −/−) pigs, which, while on a high fat diet, develop human-like coronary plaques^22^.

In the present study, we compared the tissue reactions of coronary arteries after 3rd EES and 2nd EES stenting, from autopsy cases as well as in LDLR −/− minipigs.

## Results

### Human autopsy cases

Atherosclerotic risk factors did not differ between the groups receiving 3rd EESs (Synergy, Boston Scientific, Natick, MA, USA) and 2nd EESs (Xience, Abbott Vascular, Abbott Park, IL, USA) (Supplementary Table 1). The medical history, including cause of death, risk factors, cardiovascular complications, and stent implantation period, is presented for each case (Supplementary Table 2). None of the cases enrolled in this study showed ST. Stent details including number of stents, number of histological sections, number of struts, stent implanted period, lengths and diameters of stents, and the location of the vessel in which the stent was implanted are shown in Table 1. Stent characteristics differed minimally between the two groups.

**Table 1:** Stent data of human autopsy cases.

	Third-generation EES n (number of cases) = 4	Second-generation EES n (number of cases) = 9	*p* value
Number of stents	7	12	–
Number of stent struts (histology)	142	389	–
Stent implanted period (months)	4.8 ± 0.7	5.4 ± 0.9	0.717
Stent length (mm)	26.0 ± 2.7	23.8 ± 1.8	0.232
Stent diameter (mm)	2.68 ± 0.1	3.02 ± 0.1	0.093
Right coronary artery	0 (0%)	4 (33%)	–
Left anterior descending artery	4 (57%)	7 (58%)	–
Left circumflex artery	3 (43%)	1 (8%)	–

### Histological evaluation of the human autopsy cases

**Figure 1:**
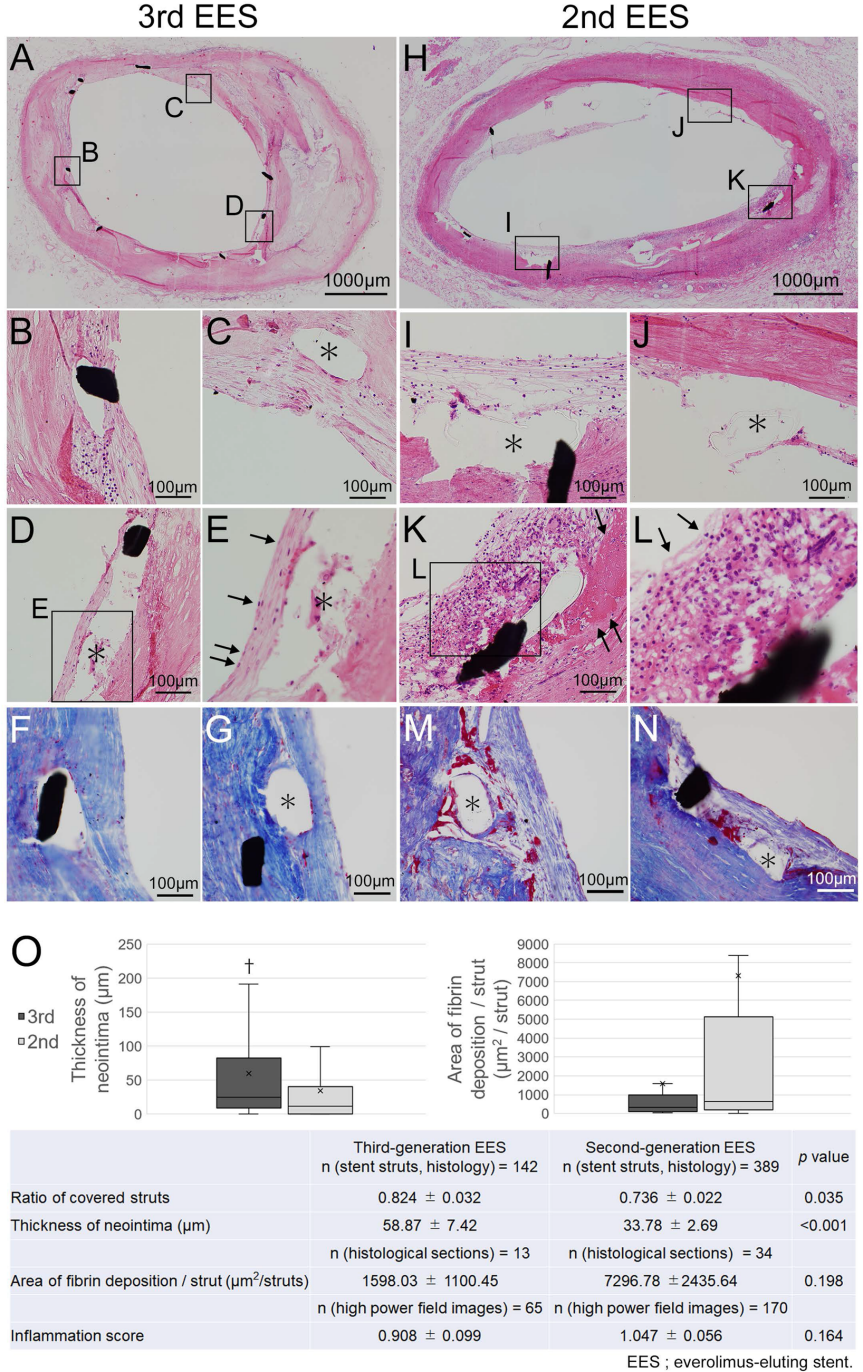
Pathology of human autopsy cases. (**A**–**N**) Representative images of human coronary arteries with implanted third-generation abluminal biodegradable-polymer everolimus-eluting stents (3rd EES, **A**–**G**) and second-generation durable-polymer everolimus-eluting stents (2nd EES, **H**–**N**). (**A**) Most struts of the 3rd EES are covered with neointima. (**B**–**D**) The neointima after 3rd EES implantation shows a well-developed matrix with tightly arranged smooth muscle cells. There is no infiltration of inflammatory cells. (**E**) The luminal surface is well-covered by endothelial cells (arrows). (**F**, **G**) Few fibrin deposits are observed around the 3rd EES struts. (**H**, **J**) Some struts of the 2nd EES are not covered. (**I**) Neointima after 2nd EES implantation consists of loose connective tissue. (**K**) Advanced inflammatory cell infiltration and fibrin deposits (arrows) are seen around the struts of the 2nd EESs. (**L**) Advanced inflammatory cell infiltration and neointima partially lacking endothelial cells (arrows) are shown. (**M**, **N**) Fibrin deposition is prominent around the 2nd EES struts. (**O**) Morphometrical analysis of the 3rd and the 2nd EES tissues. Although both 3rd and 2nd EESs show a high degree of coverage, the 3rd EES has better coverage. †*p* < 0.05. (**C**–**E**, **G**, **I**, **J**, **M**, **N** *; stent strut. **A**–**E**, **H**–**L**; hematoxylin–eosin. **F**, **G**, **M**, **N**; Masson’s trichrome).

**Figure 2:**
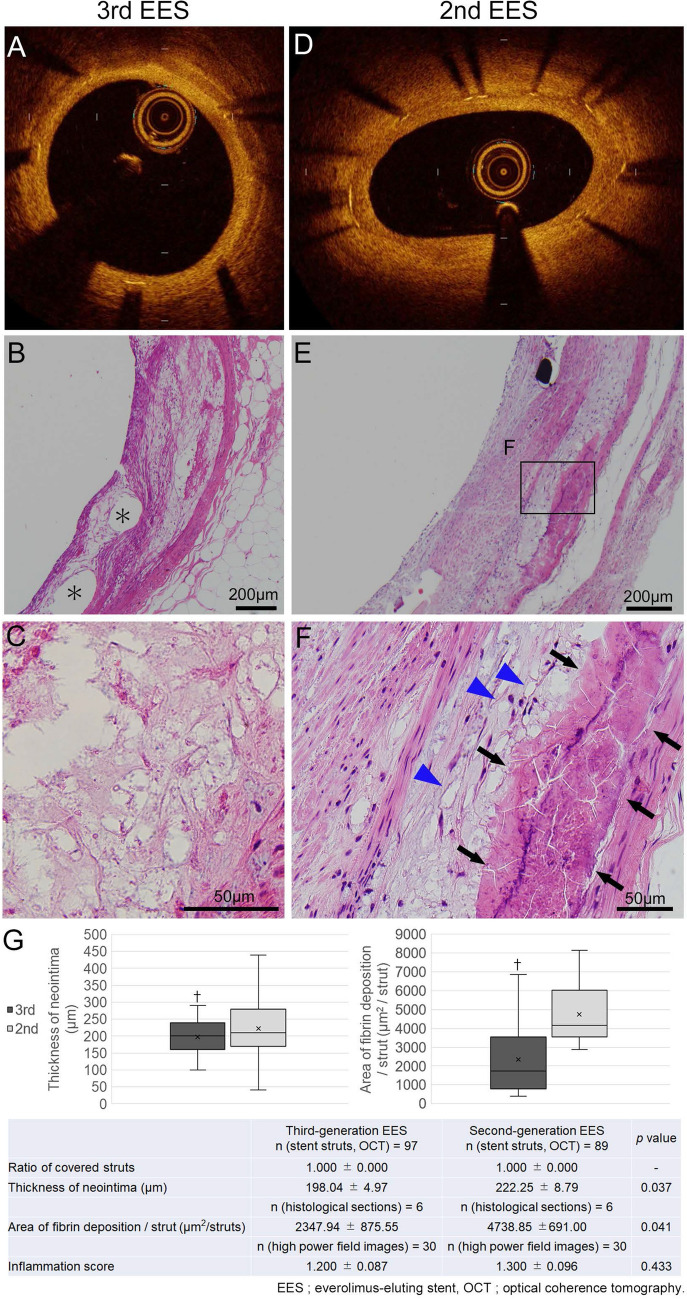
Optical coherence tomography images and pathology of the 4-week stent lipoprotein receptor knockout minipigs. Representative images from the 4-week group of coronary arteries of lipoprotein receptor knockout minipigs implanted with third-generation abluminal biodegradable-polymer everolimus-eluting stents (3rd EES, **A**–**C**) and second-generation durable-polymer everolimus-eluting stents (2nd EES, **D**–**F**). (**A**, **D**) On optical coherence tomography, all struts of both 3rd and 2nd EESs were shown to acquire adequate neointimal coverage. Histological sections of coronary arteries implanted with 3rd (**B**, **C**) and 2nd (**E**, **F**) EESs. Underlying atherosclerotic change with extracellular lipid accumulation (**C**), foam cell infiltration (arrowheads in **F**) and calcification (arrows in **F**) are apparent. The neointima of the 3rd EES showed tightly arranged smooth muscle cells close to the luminal surface (**B**) while the neointima over 2nd EESs consisted of poorly structured connective tissue (**E**). (**G**) There is significantly less neointimal thickening and fewer fibrin depositions with the 3rd EES than with the 2nd EES. †*p* < 0.05 (**B** *; stent strut. **B**, **C**, **E**, **F**; hematoxylin–eosin).

Ratios of covered stent struts for 3rd EESs (Fig. 1A) and 2nd EESs (Fig. 1H,J) were 0.824 ± 0.032 and 0.736 ± 0.022 (*p* = 0.035), respectively. In addition, the neointima was significantly thicker over 3rd EESs than over 2nd EESs (58.87 ± 7.42 µm vs. 33.78 ± 2.69 µm, *p* < 0.001) (Fig. 1O). The lumen area was adequately maintained without restenosis in both groups.

After the 3rd EESs implantation, the neointima showed a well-developed matrix with relatively tightly arranged smooth muscle cells (SMCs) (Fig. 1B–D). In addition, the luminal surface was well-covered by endothelial cells (Fig. 1E, arrows) and fibrin deposition was minimal (Fig. 1F,G). On the other hand, the neointima over 2nd EESs consisted of poorly structured connective tissue (Fig. 1I). Furthermore, several cases had inflammatory cell infiltration and fibrin deposition around the struts (Fig. 1K–N), though these reactions were heterogenous. Part of the neointima lacked endothelial cells (arrows in 1L). As to fibrin deposition, no significant difference was seen between the two stent types (3rd EES: 1598.03 ± 1100.45 µm^2^/strut vs. 2nd EES: 7296.78 ± 2435.64 µm^2^/strut, *p* = 0.198). In addition, the inflammation score also did not show significant difference (3rd EES: 0.908 ± 0.099 vs. 2nd EES: 1.047 ± 0.056, *p* = 0.164).

## Coronary arteries in LDLR −/− minipigs after stent implantation

Data on stenting such as the number of stents, number of sections assessed by optical coherence tomography (OCT), number of struts detected by OCT, lengths and diameters of the stents, and the location of the vessel in which the stent was implanted, for the 4-week follow-up group (4w group) and the 2-week follow-up group (2w group) are summarized in Table 2 and Supplementary Table 3, respectively.

### OCT evaluation of the animal model

In the 4w group of LDLR −/− minipigs, stent struts of 3rd EESs (Fig. 2A) and 2nd EESs (Fig. 2D) were both entirely covered. The thicknesses of the neointima over 3rd EESs and 2nd EESs were 198.04 ± 4.97 µm vs. 222.25 ± 8.79 µm for the 4w group (*p* = 0.037). Additionally, in the 2w group of LDLR −/− minipigs, ratios of covered struts were 0.795 ± 0.047 for 3rd EESs (Supplementary Fig. 1A) and 0.788 ± 0.050 for 2nd EESs (Supplementary Fig. 1E) (*p* = 0.926). The thicknesses of the neointima over 3rd EESs and 2nd EESs were 43.29 ± 3.29 µm vs. 61.67 ± 5.51 µm (*p* = 0.009). The neointima of 3rd EESs was significantly thinner than that of 2nd EESs for the first 4 weeks after stent implantation (Fig. 2G, Supplementary Fig. 1I).

### Histological analysis of the animal model

Confirming the status of coronary arteries in which stents had been implanted, underlying atherosclerotic changes, such as extracellular lipid accumulation (Fig. 2C), foam cell infiltration (Fig. 2F, arrowheads), and calcification (Fig. 2F, arrows) were seen.

The neointima of the 3rd EES showed tightly arranged SMCs close to the luminal surface (Fig. 2B) while the neointima over 2nd EESs consisted of poorly structured connective tissue (Fig. 2E). The neointima of the 2w group was thinner for the 3rd than the 2nd EES (Supplementary Fig. 1B, F). SMCs were tightly packed, and peri-strut inflammatory cell infiltration and fibrin deposition were insignificant after 3rd EES implantation (Supplementary Fig. 1C and D). On the contrary, after 2nd EES implantation, aggregation of inflammatory cells was observed adjacent to the luminal sides of struts (Supplementary Fig. 1G and H, arrows).

There was significantly less fibrin deposition around the struts in the 3rd EESs than in the 2nd EESs in both the 4w group (2347.94 ± 875.55 µm^2^ vs. 4738.85 ± 691.00 µm^2^, *p* = 0.041) (Fig. 2G) and the 2w group (669.45 ± 199.05 µm^2^ vs. 1681.32 ± 430.38 µm^2^, *p* = 0.044) (Supplementary Fig. 1I). The inflammation score did not differ significantly in the 4w group (3rd EES: 1.200 ± 0.087 vs. 2nd EES: 1.300 ± 0.096, *p* = 0.433) (Fig. 2G). In this regard, we also note that 3rd EES implantation (Supplementary Fig. 1B–D) appeared to rarely induce the inflammatory response seen with 2nd EES implantation (Supplementary Fig. 1F–H) at 2 weeks (0.967 ± 0.078 vs. 1.509 ± 0.106, *p* < 0.001) (Supplementary Fig. 1I).

**Figure 3:**
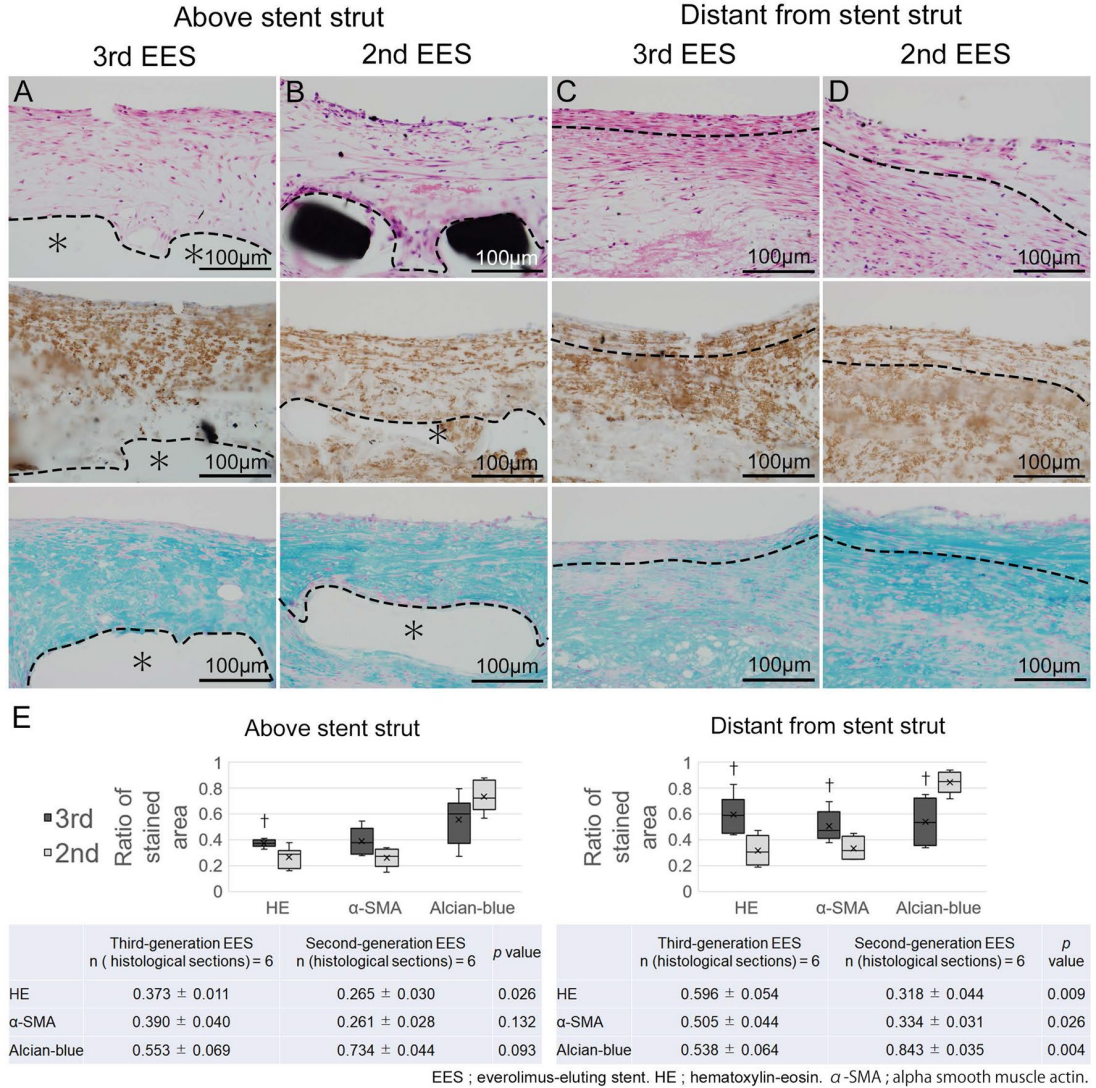
Neointimal tissue characteristics of the 4-week stent implantation group of low-density lipoprotein receptor knockout minipigs. (**A**–**D**) High-power magnification images of the neointima from the 4-week group of coronary arteries of low-density lipoprotein receptor knockout (LDLR −/−) minipigs implanted with the third-generation abluminal biodegradable-polymer everolimus-eluting stents (3rd EES, **A**, **C**) and second-generation durable-polymer everolimus-eluting stents (2nd EES, **B**, **D**) (upper panel: Hematoxylin–eosin, middle panel: alpha-Smooth muscle actin, lower panel; acian-blue). (**A**, **B**) Show the neointima above stent struts and (**C**, **D**) show the neointima distant from the stent struts. The part above the dotted line corresponds to the neointima and the part below to the intima and media. (A, **C**) Neointima after the 3rd EES shows several compact layers of tightly arranged smooth muscle cells (SMCs), close to the luminal surface. (**B**, **D**) Neointima after the 2nd EES. Although thicker than that seen after the 3rd EES implantation, the neointima consists of proteoglycan-rich tissue and its density of SMCs is relatively low. (**E**) Neointima above the stent struts of the 3rd EES demonstrated significantly higher SMC density by hematoxylin–eosin staining than those with the 2nd EES. Neointima distant from the stent struts of the 3rd EES shows statistically significant differences with higher SMC and a lower density of alcian-blue-positive proteoglycans. †*p* < 0.05. (**A**, **B** *; stent struts).

**Figure 4:**
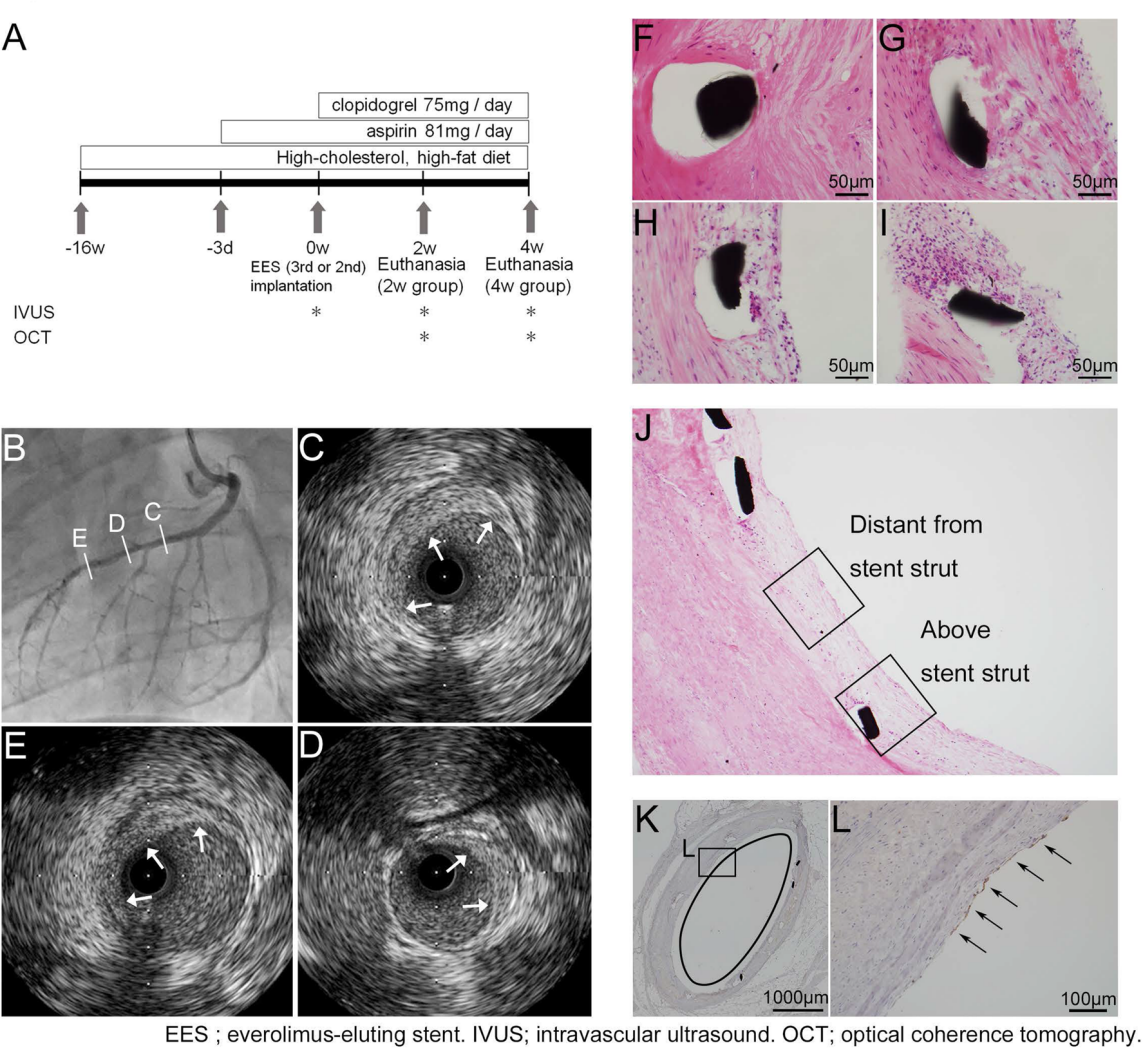
Protocols for animal experimentation and morphometrical analysis. (**A**) Outline of low-density lipoprotein receptor knockout (LDLR −/−) minipigs experiment. (**B**–**E**) Images of coronary arteries from LDLR −/− minipigs before stent implantation. (**B**) Angiographically, mild stenosis due to arteriosclerosis is visible. (**C**–**E**) Eccentric fibro-fatty plaques (arrows) are observed on intravascular ultrasound. (**F**–**I**) Grading of peri-strut inflammation for semi-quantitative analysis. (**F**) Grade 0, absent, few inflammatory cells. (**G**) Grade 1, mild, fewer than 20 inflammatory cells per high power field (HPF). (**H**) Grade 2, moderate, fewer than 50 inflammatory cells per HPF. (**I**) Grade 3, severe, more than 50 inflammatory cells per HPF. (**J**) Tissue characteristics are evaluated for the neointima above the stent strut and the neointima distant from the stent struts. (**K**, **L**) All sections of stented regions harvested from porcine coronary arteries are immunostained for anti-CD31 antibody. The total luminal circumference (**K**) was measured and the lengths of re-endothelialized regions (arrows in **L**) were added up. The ratio of the re-endothelialized region was calculated (**F**–**J**; hematoxylin–eosin, **K**, **L**; immunohistochemistry for anti-CD31 antibody. **C**, **D** *; stent strut).

Characteristics of neointimal tissue were also evaluated in the 4w group (Fig. 3). The neointima just above the stent struts (Fig. 4J) of a 3rd EES demonstrated significantly elevated SMC density on morphometrical analysis using hematoxylin–eosin. The alcian-blue staining and immunohistochemistry for alpha-smooth muscle actin (α-SMA) showed no differences between the two stent types (Fig. 3A,B). Neointima distant from the stent struts (Fig. 4J) of the 3rd EESs showed several SMC layers with mature extracellular matrix (Fig. 3C), while that of 2nd EESs showed a lower density of SMC with abundant alcian-blue-positive proteoglycans (Fig. 3D). The morphometrical and statistical data are summarized in Fig. 3E.

The degree of re-endothelialization did not differ between 3rd EESs and 2nd EESs in either the 4w (3rd EES: 0.439 ± 0.045 vs. 2nd EES: 0.432 ± 0.038, *p* = 0.818) or the 2w (3rd EES: 0.257 ± 0.052 vs. 2nd EES: 0.290 ± 0.098, *p* = 1.000) group, despite the differences in neointimal thickness and structure between the two stent types.

## Discussion

Since the development of DES, complications such as VLST and neoatherosclerosis, due to delayed arterial healing, have caused serious problems^3,4,23–25^. As shown in the present study, 3rd EES implantation in human coronary arteries achieves in a healing process with greater coverage of struts than 2nd EES implantation.

We previously reported the healing process after the 1st DES implantation to be limited. Within 1 year after 1st DES implantation, only 44% of the struts were covered by neointima^14^. In the current study, both 2nd and 3rd EESs showed a higher degree of strut coverage than the 1st DES. Furthermore, a comparison between 2nd and 3rd EESs revealed the latter to show a significantly higher proportion of covered struts.

There was no significant difference between the two EESs in fibrin deposition around stent struts. This lack of a statistically meaningly difference might be attributable to the heterogeneity of the sites at which stents had been implanted, such as various anatomical location, luminal diameters, three-dimensional structures, and blood flow effects. However, statistically significant differences may emerge as more cases are accumulated.

In coronary arteries of LDLR −/− pigs, both 3rd and 2nd EESs acquired sufficient neointimal coverage relatively soon after implantation. Although no significant difference in the degree of strut coverage between the two EESs was seen, their histological characteristics were clearly different.

In the 4w group, neointimas after 2nd EES implantation were composed of proteoglycan-rich tissue. In contrast, over the 3rd EES struts, SMCs were arranged in several compact layers close to the luminal surface. Our speculation, based on the additional data for the 2w group, is that the absence of drugs on the luminal side of the 3rd EESs allows SMC proliferation without inducing inflammatory cell infiltration and fibrin deposition.

Two weeks after implantation, the 3rd EESs showed less inflammation and fibrin deposition around stent struts. The lower inflammatory response observed around the struts of 3rd EESs might be attributable to the design of these EESs, which are coated with polymer only on the side that is in contact with the intima, while the 2nd EESs are circumferentially coated with the polymer. Inflammation, particularly that seen on the luminal side of the struts of 2nd EESs, suggests that this is a response to the polymers coating the luminal side. In the 2nd EESs, maturation of the neointimal tissue appeared to be inhibited by the drug released from the polymer on the luminal side of the struts and/or the polymer itself. As to the fibrin deposition found around stent struts, we speculate that the difference in the dimensions of the struts of 3rd and 2nd EESs may be related. Differences in the geometric structures of struts are reportedly involved in thrombus formation^26^. The struts of the 2nd EESs are thicker than those of the 3rd EESs (2nd EES: 81 µm vs. 3rd EES: 74 µm), and may thus account for the difference in the amount of fibrin deposition. In addition, inflammation, as observed around 2nd EES struts, may have induced fibrin deposition. Indeed, inflammatory and thrombotic pathways have been described as sharing signaling pathways, thereby connecting inflammatory responses to the clotting cascade^27^.

The main functions of endothelial cells are regulation of vascular tone, fluid filtration, neutrophil recruitment, hormone trafficking, and hemostasis, as well as suppressing inflammation and thrombosis^28–32^. Since drugs eluted by DES suppress proliferation of not only SMCs but also endothelial cells, they are known to underlie the occurrence of VLST and the formation of neoatherosclerosis. Thus, early restoration of endothelium is important after percutaneous coronary intervention procedures. The 3rd EES was designed using abluminal polymers intended to reduce the suppression of re-endothelialization. However, our study in LDLR −/− minipigs demonstrated no improvement in re-endothelialization of 3rd EESs as compared with 2nd EESs, which is in line with the results of a previous study^33^.

After 3rd EES implantation in LDLR −/− minipigs, the neointima required less time to become well-developed, as compared to 2nd EES implantation. Inflammatory responses and fibrin deposition were more prominent in 2nd EESs, and may underlie the observed delay in arterial healing.

There is clearly a discrepancy in neointimal thicknesses between humans and the porcine model. While the neointima was found to be significantly thicker for 3rd EESs in human autopsy cases, the opposite was noted in pigs. We attribute these contradictory results to the difference in healing reactions, particularly as regards neointimal proliferation, between human and porcine tissues. Healing reaction processes are more rapid in pigs than in humans, namely the phenomenon of neointima formation, as shown by both generations of stent struts implanted in porcine coronary arteries being completely covered by neointima within 4 weeks. Although we conduct our animal experiments using atherogenic pigs, the degree of atherosclerosis is more advanced in humans who are candidates for stent implantation than in this porcine model. We speculated that stent struts had been placed close to the SMC-rich media in pigs; therefore, neointimal coverage proceeded rapidly. In humans, our results indicate better healing reactions with 3rd EESs than with 2nd EESs, i.e., higher strut coverage and thicker neointima in the former. Our 3rd EES results in pigs reflect the advantages of maturation of the extracellular matrix in the neointima with less fibrin deposition and less inflammation. Although our porcine model does not entirely correspond to human pathology, the 2w and 4w LDLR −/− pig model does provide important information.

Although the time courses of tissue reactions differ between humans and LDLR −/− minipigs, the coronary artery tissue reaction against EES implantation seen in the LDLR −/− minipigs partially corresponded to the reactions observed in humans. The time courses of neointimal growth, after stent implantation, differ among species and are, as noted above, much faster in pigs than in humans^34^. In the current study, the tissue reaction seen around the EES strut of the 2w group of LDLR −/−minipigs was similar to that observed in humans with an average EES implantation period of approximately 5 months. We can speculate that the characteristics of the neointima in the 4w group of LDLR −/− minipigs might reflect the tissue response of humans beyond 1 year after stent implantation.

We suggest that these LDLR −/− minipigs may provide a model for preclinical stent development studies that closely resembles human tissue responses.

In conclusion, the results obtained from human autopsy and atherosclerotic pig experiments confirmed the clinical benefits of 3rd EES implantation. Our results indicate these stents to be associated with less inflammation and to require less time for the acquisition of a well-developed matrix than 2nd EES.

### Study limitations

Due to the relatively recent clinical introduction of the 3rd DESs for treating coronary artery disease, it was difficult to include an adequate number of cases with these newer DESs. Although we studied autopsy cases whose stents had been implanted within a year prior to death, we can speculate that tissue heterogeneity exists among autopsy cases. Due to the limited number of human autopsy cases in the current study, we could not perform a subgroup analysis for the duration of stent implantation.

There were autopsy cases in which two stents had been implanted in one coronary artery. These stents are not for the treatment of in-stent restenosis but, rather, for treating longstanding lesions. Nevertheless, it is difficult to consider these stents independently and they certainly interact with each other in terms of blood flow and other factors. In addition, one individual with lung cancer was included in the 3rd EES group. Malignancies are known to be associated with coagulation system abnormalities which might affect the formation of neointima after DES deployment. However, the presence of cancer can be also regarded as a source of the heterogeneity among human autopsy cases.

Since the duration of absorption of the polymer on the 3rd DES is 3 months, cases with longer follow-up are needed to evaluate tissue responses after complete absorption of the biodegradable polymer. We have, in fact, examined autopsy cases with stent implantation periods of more than 1 year. However, we did not include these cases in the present study since the heterogeneity among specimens from such cases is much more prominent in this population.

The number of pigs included in the experiment was, unfortunately, lower than would have been ideal. However, despite the small number of pigs, certain trends were detected in the porcine experiments. Also, in accordance with ethical guidelines for animal experimentation, we used the minimum number of animals possible.

We could not perform OCT examinations on the specimens obtained from the autopsy cases. Moreover, since we did not have the technology to remove resin after polymerization of the plastic, resin removal was not carried out for the specimens obtained from the cases autopsied before 2016. For these specimens, it was technically difficult to perform immunohistochemistry. Since 2017, removal of resin has been an option for our research group and immunohistochemistry was also available, as shown in the porcine experiment. For these reasons, it was difficult to directly compare human autopsy cases and the porcine model.

## Methods

### Approval for animal experiments

All animal care and experiments were conducted in accordance with the National Institutes of Health Guidelines for the Care and Use of Laboratory Animals (http://oacu.od.nih.gov/regs/index.htm. Eighth Edition; 2011) and the Basic Guidelines for Conduct of Animal Experiments published by the Ministry of Health, Labor and Welfare, Japan (https://www.mhlw.go.jp/shingi/2007/01/dl/s0124-6e.pdf). The study was carried out in compliance with the ARRIVE guidelines (http://www.nc3rs.org.uk/page.asp?id=1357). The experimental protocol was approved by University Gene Recombination Experiment Safety Committees (No. 2010M5) and Animal Care and Use Committees (No. AP17M048) of Nihon University School of Medicine.

### Human coronary artery specimens

The pathological specimens of coronary arteries were obtained from the autopsy cases at Nihon University Itabashi Hospital, Showa University Hospital, Hyogo College of Medicine College Hospital, Dokkyo Medical University Hospital, and Osaka Police Hospital. The experimental and ex vivo study protocols were approved by the Ethics Review Board of Nihon University Itabashi Hospital (RK-171212-6). Written informed consent from the bereaved family was obtained for the research use of specimens. The autopsies were performed according to the guidelines of the institutes mentioned above. Information on the medical histories of the patients, including any medications, were obtained from medical records. In order to evaluate the early tissue response after DES implantation without including acute cases, specimens obtained from cases autopsied 1–12 months after implantation of 3rd EESs or 2nd EESs for stable angina pectoris treatment were included in the current study. Specimens obtained from the cases autopsied more than 1 year after stent implantation were excluded. Cases that underwent stent implantation for clinical conditions including plaque rupture and thrombosis associated with unstable angina pectoris were excluded. Specimens from acute and recent myocardial infarction cases were also excluded. In addition, lesions with overlapping stents due to additional stent implantation for in-stent restenosis were excluded. The autopsies had been performed during the period from 2012 through 2019.

Pathological specimens were prepared employing the following procedure. Coronary arteries were removed from the heart at autopsy within 6 h after death, and whole stent segments were fixed in 10% buffered formalin for 48 h and obtained decalcification in 10% ethylenediaminetetraacetic acid disodium solution for 48 h. Samples containing struts were embedded in plastic according to the guidelines of the manufacturer (Osteoresin Embedding Kit, FUJIFILM Wako Pure Chemical Corporation, Osaka, Japan). After polymerization of the plastic, stent segments were cut at approximately 5 mm intervals and histologic sections were prepared at 5 µm with a tungsten carbide knife. Then, staining with hematoxylin–eosin, Masson’s trichrome, and elastica van Gieson was performed. Seven stents from 4 autopsy cases with 3rd EESs (stent implanted period of 3–7 months) and 12 stents from 9 autopsy cases with 2nd EESs (stent implanted period of 1–10 months) were examined. Clinical backgrounds of patients and stent characteristics are shown in Supplementary Tables 1 and 2, and in Table 1.

### Animal preparation and procedures

The LDLR −/− minipigs with human-like advanced coronary plaque development used in this study were previously described in detail by Li et al.^22^. All animals were housed at the Nihon University Medical Research Support Center, and standard animal husbandry procedures were followed.

To accelerate the development of coronary artery plaques, 3-month-old male LDLR −/− minipigs (n (number of animals) = 6) were fed a high-cholesterol, high-fat (HCHF) diet (1 kg/day) throughout the experiment (Fig. 4A). The HCHF diet was made by adding 1.5% cholesterol and 15% powdered fat (Feed one Co., Ltd, Kanagawa, Japan) to standard pig chow.

After feeding of the HCHF diet for 4 months, the pigs were anesthetized with 3% sevoflurane. Using a general sterile technique, a 6F vascular sheath was inserted through the left carotid artery and heparin (100 IU/kg) was injected before catheterization. Angiography of the coronary arteries was performed (Fig. 4B). Guided by intravascular ultrasound (IVUS), pigs were stented in randomly selected coronary arteries with a durable-polymer PROMUS Premier EES (Boston Scientific) for the 2nd DES and biodegradable-polymer Synergy EES for the 3rd DES. For IVUS, iLab System Cart with Opticross Imaging Catheter (Boston Scientific) was used (Fig. 4C–E). The stents were deployed in the location of the most intense atherosclerosis within the coronary artery. At the selected location, the balloon was inflated to achieve a stent-to-artery ratio of 1.1:1. All pigs were orally given aspirin (81 mg/day) 3 days prior to stent deployment. After stenting, the animals received clopidogrel (75 mg/day) until the day of sacrifice. Four weeks (n (number of animals) = 4) after stent implantation, coronary angiography, IVUS and OCT were performed in all stented vessels. For OCT, OPTIS Mobile Imaging Software with Dragonfly OPTIS Imaging Catheter (Abbott, Santa Clara, CA, USA. https://www.cardiovascular.abbott/us/en/hcp/products/percutaneous-coronary-intervention/optis-integrated-system-intravascular-imaging.html) was used for observation of the intravascular condition. The ratio of struts covered by neointima and neointimal thicknesses were evaluated using OCT images. Three OCT slices were randomly selected from the distal, middle, and proximal part of each stent. The number of stent struts and the struts with neointimal coverage were counted, and the neointimal thickness for each stent strut was digitally measured. Struts were defined as being covered when the neointimal thickness was at least 0.04 mm, since it was difficult to recognize neointimal coverage on OCT when the neointima was thin, i.e. less than 0.03 mm.

The pigs were sacrificed immediately after OCT examination. The coronary arteries were removed from the heart and whole stent segments were fixed in 10% buffered formalin for 48 h and obtained decalcification in 10% ethylenediaminetetraacetic acid disodium solution for 48 h, then embedded in plastic with the strut. After polymerization of the plastic, stents were segmented at 3–5 mm intervals and histologic sections were cut at 5 µm with a tungsten carbide knife. The specimens were stained with hematoxylin–eosin, Masson’s trichrome, elastica van Gieson, or alcian-blue. In addition, immunohistochemistry was performed with a rabbit polyclonal anti-CD31 antibody (#ab28364, 1:20, Abcam, Cambridge, UK) and a mouse monoclonal anti-α-SMA antibody (#M0851, 1:4000, Dako, Agilent, Santa Clara, CA). Histological sections that had been damaged during the cutting or staining procedures were excluded.

OCT data and histopathology of the 4w group showed rapid neointimal growth, possibly reflecting the tissue response of humans beyond 1 year after stent implantation. Therefore, we added a study of the 2w group (n (number of animals) = 2) to investigate earlier tissue reactions in the animal model. Details on the number and location of stents for each experimental group are presented in Table 2 and Supplementary Table 3.

**Table 2:** Stent data of 4-week follow-up low-density lipoprotein receptor knockout minipigs.

	Third-generation EES	Second-generation EES
Number of stents	4	4
Number of OCT sections	46	46
Number of stent struts (OCT)	97	89
Stent length (mm)	20.0 ± 0.0	20.0 ± 0.0
Stent diameter (mm)	3.1 ± 0.1	3.3 ± 0.1
Right coronary artery	2 (50%)	1 (25%)
Left anterior descending artery	2 (50%)	2 (50%)
Left circumflex artery	0 (0%)	1 (25%)

### Histological assessments

For human autopsy cases, the numbers of covered and total stent struts in each section were counted to calculate the ratio of struts covered by neointima. We considered stent struts to be covered if there was a microscopically detectable neointima over the struts. The neointimal thickness of the stent struts was defined as the distance from the margin of the struts to the luminal surface and was measured by image analysis software, ImageJ^35^ (U. S. National Institutes of Health, Bethesda, Maryland, USA). For all histological sections, the area of fibrin deposition and the inflammation scores were evaluated. Area of fibrin deposition, defined as a reddish area around the stent struts on Masson’s trichrome staining, was measured by ImageJ. The area of fibrin deposition was divided by the number of stent struts in each section, in order to facilitate comparison between the sections. Inflammation scores around the struts were semi-quantitatively graded by observation of 5 high power fields (HPFs) per section. Scores were defined as follows: Grade 0, absent, few inflammatory cells except some infiltrating foreign body giant cells; Grade 1, mild, no more than 20 inflammatory cells; Grade 2, moderate, no more than 50 inflammatory cells; Grade 3, severe, more than 50 inflammatory cells per HPF (Fig. 4F–I). The maturity of neointima was evaluated in specimens harvested from the 4w group of LDLR −/− minipigs. Mature neointima is considered to contain SMCs, which are eosin- and α-SMA-positive, while immature neointima is largely composed of extracellular matrix, containing proteoglycans which are alcian-blue-positive, with a few SMCs^36^. The assessment was performed for the neointima above the stent struts and that distant from the stent struts (Fig. 4J). We randomly selected one HPF of the neointima above the stent struts and one distant from the stent struts per section. The ratio of the eosin-stained area per neointimal area was calculated using ImageJ. Simultaneously, the ratios of the α-SMA-stained area and the alcian-blue stained area per neointimal area were calculated in the same way to evaluate the presence of SMCs and deposition of proteoglycans, respectively. Observation of Masson’s trichrome clearly indicates the difference in extracellular matrix maturity, as compared to hematoxylin–eosin, at the area adjacent to the stent struts. The mature matrix is in the intima, while the immature matrix is in the neointima. Although identification of the border between the intima and neointima can be challenging in the area distant from the struts (Figs. 3C and 4D), we traced the border between the intima and neointima close to the struts to identify the border between them. Evaluation of neointimal tissue characteristics was only applied to specimens from the 4w group, since the neointima observed in specimens harvested from the 2w group was too thin. Also, some struts remained uncovered. On the other hand, the degree of re-endothelialization of the neointima was evaluated for both the 2w and the 4w group. Lumen length and the region covered by endothelial cells, which are immunohistochemically CD31-positive, were measured for each section (Fig. 4K, L). The ratio of the re-endothelialized region was calculated by the following formula: length of re-endothelialized region/lumen length.

Since the autopsy cases were from multiple institutes, the pathologists performing the autopsies differed among cases. However, information on the medical histories of the patients, including any medications, was consistently retrieved from their autopsy reports and medical records, and all of the measurements and assessments of histological sections were performed by one experienced evaluator (S.M.).

### Statistical analysis

Results are presented as the means ± SEMs for continuous variables. For statistical analysis, differences between groups were evaluated using the chi-square of the Fisher exact test (for categorical variables), and the t-test (for continuous variables). We used the Shapiro–Wilk normality test for assessing the normality of distributions for the morphometrical analysis. Not all of the assessment categories had a normal distribution in the current study. Thus, we used the Mann–Whitney test for morphometrical analysis. We considered *p* < 0.05 to indicate a statistically significant difference. All statistical analyses were performed with EZR (Saitama Medical Center, Jichi Medical University, Saitama, Japan), which is a graphical user interface for R (The R Foundation for Statistical Computing, Vienna, Austria). More precisely, it is a modified version of R commander designed to add statistical functions frequently used in biostatistics^37^.

## Supplementary Information

Supplementary Information

## Data Availability

The datasets generated and/or analyzed in the current study are available from the corresponding author on reasonable request.
