# Supplementary material for: Pathological findings after third- and second-generation everolimus-eluting stent implantations in coronary arteries from autopsy cases and an atherosclerotic porcine model
Source: Sci Rep. 2021 Mar 18;11:6281. doi: 10.1038/s41598-021-85740-2 (PMC7973802; doi:10.1038/s41598-021-85740-2)
Supplement: Supplementary file 1 — Supplementary Information [file 41598_2021_85740_MOESM1_ESM.docx]

**Pathological Findings After Third- and Second-Generation Everolimus-Eluting Stent Implantations in Coronary Arteries from Autopsy Cases and an Atherosclerotic Porcine Model**

Suguru Migita^1,2^; Daisuke Kitano^2^; Yuxin Li^3^*; Yutaka Koyama^1,2^; Sayaka Shimodai-Yamada^1^; Akira Onishi^4^; Daiichiro Fuchimoto^5^; Shunichi Suzuki^5^; Yoshiyuki Nakamura^6^; Taka-aki Matsuyama^7^; Seiichi Hirota^8^; Masashi Sakuma^9^; Masahiko Tsujimoto^10^; Atsushi Hirayama^11^; Yasuo Okumura^2^; Hiroyuki Hao^1^*

^1^Division of Human Pathology, Department of Pathology and Microbiology, Nihon University School of Medicine, Tokyo, Japan.

^2^Division of Cardiology, Department of Medicine, Nihon University School of Medicine, Tokyo, Japan.

^3^Division of Cell Regeneration and Transplantation, Department of Functional Morphology, Nihon University School of Medicine, Tokyo, Japan.

^4^Department of Animal Science and Resources, Nihon University, College of Bioresource Sciences, Kanagawa, Japan.

^5^Institute of Agrobiological Sciences, National Agriculture and Food Research Organization (NARO), Ibaraki, Japan.

^6^Saitama Prefectural Agricultural Technology Research Center, Swine and poultry research, Saitama, Japan.

^7^Department of Legal Medicine, Showa University School of Medicine, Tokyo, Japan.

^8^Department of Surgical Pathology, Hyogo College of Medicine, Nishinomiya, Japan.

^9^Department of Cardiovascular Medicine, Dokkyo Medical University School of Medicine, Tochigi, Japan.

^10^Department of Pathology and ^11^Cardiology, Osaka Police Hospital, Osaka, Japan.

*Corresponding author: Hiroyuki Hao, M.D., Ph.D., 30-1 Oyaguchi-kamicho, Itabashi-ku, Tokyo 173-8610, Japan. TEL: +81-3-3972-8111, FAX: +81-3972-8830, E-mail: [hao.hiroyuki@nihon-u.ac.jp](about:blank)

Yuxin Li, 30-1 Oyaguchi-kamicho, Itabashi-ku, Tokyo 173-8610, Japan. TEL: +81-3-3972-8111, FAX: +81-3972-8666 E-mail: [li.yuxin@nihon-u.ac.jp](about:blank)

**Supplementary Table 1. Clinical background of human autopsy cases.**

|  | Third-generation EES  n (number of cases) = 4 | Second-generation EES  n (number of cases) = 9 | *p* value |
| --- | --- | --- | --- |
| Age (years) | 68.7 ± 3.5 | 78.3 ± 2.3 | 0.279 |
| Male | 3 (75%) | 9 (100%) | 0.118 |
| Hypertension | 4 (100%) | 4 (44%) | 0.057 |
| Diabetes mellitus | 2 (50%) | 7 (78%) | 0.317 |
| Dyslipidemia | 3 (75%) | 6 (67%) | 0.764 |
| Smoking | 2 (50%) | 3 (33%) | 0.569 |
| Hemodialysis | 1 (25%) | 1 (11%) | 0.522 |

**Supplementary Table 2. Medical history of human autopsy cases.**

|  | Sex | Age | Cause of death | Risk factors | Cardiovascular complications | Stent implanted period (months) |
| --- | --- | --- | --- | --- | --- | --- |
| Third-generation EES | | | | | | |
| 1 | M | 63 | Aortic aneurysm rupture | HT, DM, DLP | OMI, Af, AD | 7 |
| 2 | M | 77 | Myocardial infarction | HT, DLP, smoking | OMI, HD | 5 |
| 3 | M | 66 | Lung cancer | HT, DM, smoking | none | 4 |
| 4 | F | 85 | Heart failure | HT, DLP | AS, ASO, LVH | 3 |
| Second-generation EES | | | | | | |
| 1 | M | 72 | Sepsis, pancreatitis | HT, DM, smoking | OMI, CE | 6 |
| 2 | M | 74 | Myocardial infarction | HT, smoking | OMI | 2 |
| 3 | M | 81 | Aortic aneurysm rupture | DM, DLP | CI, CA stenosis, CE | 4 |
| 4 | M | 80 | Sepsis, pneumonia | HT, DM, DLP | OMI, CABG | 9 |
| 5 | M | 91 | Pneumonia | HT, DLP | CI | 2 |
| 6 | M | 78 | Heart failure | DM, DLP | OMI, CABG, HD | 1 |
| 7 | M | 76 | Interstitial pneumonia, Heart failure | DM, DLP, smoking | LVH | 10 |
| 8 | M | 86 | Alveolar hemorrhage | DM | none | 9 |
| 9 | M | 67 | Heart failure | DM, DLP | DCM, OMI | 7 |
| EES, everolimus-eluting stent; HT, hypertension; DM, diabetes mellitus; DLP, dyslipidemia; OMI, old myocardial infarction; Af, Atrial fibrillation; AD, aortic dissection; HD, hemodialysis; AS, aortic valve stenosis; ASO, arteriosclerosis obliterans; LVH, left ventricular hypertrophy; CE, cholesterol emboli; CI, cerebral infarction; CA, carotid artery; CABG, coronary artery bypass grafting; DCM, dilated cardiomyopathy. | | | | | | |

**Supplementary Table 3. Stent data of 2-week follow-up** **low-density lipoprotein receptor knockout minipigs.**

|  | Third-generation EES | Second-generation EES |
| --- | --- | --- |
| Number of stents | 2 | 2 |
| Number of OCT sections | 37 | 35 |
| Number of stent struts (OCT) | 73 | 66 |
| Stent length (mm) | 20.0 ± 0.0 | 20.0 ± 0.0 |
| Stent diameter (mm) | 3.3 ± 0.2 | 3.3 ± 0.2 |
| Right coronary artery | 1 (50%) | 1 (50%) |
| Left anterior descending artery | 0 (0%) | 0 (0%) |
| Left circumflex artery | 1 (50%) | 1 (50%) |

Fi


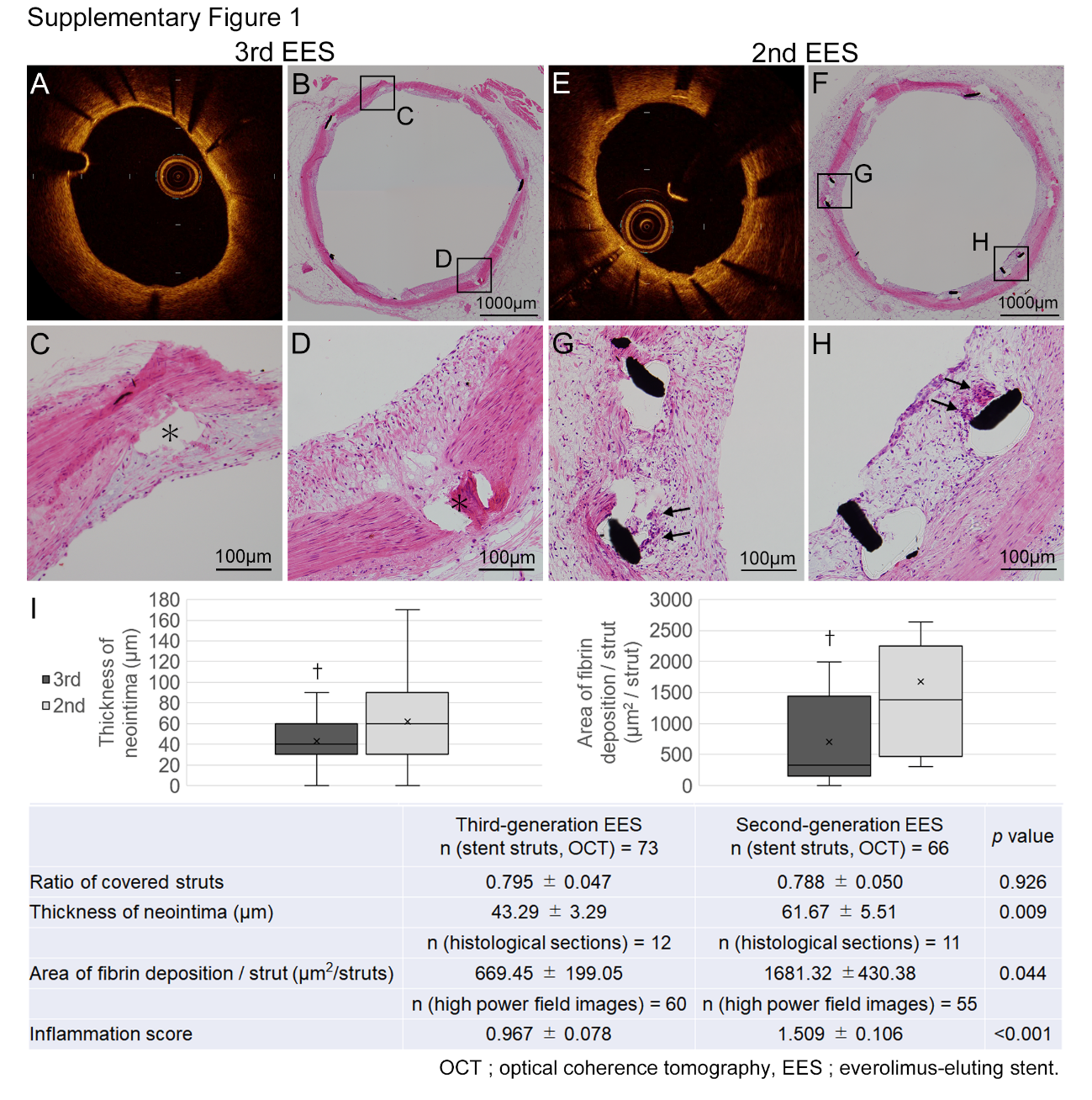


**Figure legend:**

**Supplementary Figure 1. Optical coherence tomography and pathology of the 2-week stent implantation group of low-density lipoprotein receptor knockout minipigs**

(A-H) Representative images from the 2-week group of low-density lipoprotein receptor knockout minipig coronary arteries implanted with third-generation abluminal biodegradable-polymer everolimus-eluting stents (3rd EES, A-D) and second-generation durable-polymer everolimus-eluting stents (2nd EES, E-H). On optical coherence tomography (OCT) images, all struts of both 3rd and 2nd EESs show neointimal coverage. The neointima over the 3rd EES is thinner than that over the 2nd EES (A, E). Low- (B, F) and high- (C, D, G, and H) power magnification cross sections. (B-D) Smooth muscle cells are packed tightly, and peri-strut inflammatory cell infiltration and fibrin deposition are both minimal in the neointima after the 3rd EES implantation. (F-H) Aggregations of inflammatory cells (arrows in G and H) are located just above the struts in the neointima after the 2nd EES implantation. (I) The inflammation score of the 3rd EESs is significantly lower than that of the 2nd EESs. †*p* < 0.05. (C, D ＊; stent strut. B-D, F-H; hematoxylin-eosin.)
